# Vasopressin versus norepinephrine for the management of septic shock in cancer patients

**DOI:** 10.1186/cc13351

**Published:** 2014-03-17

**Authors:** C Zambolim, D Nagaoka, J Fukushima, C Park, J Carneiro, E Osawa, J Almeida, S Zefferino, F Galas, L Hajjar

**Affiliations:** 1 Instituto do Cancer do Estado de São Paulo, Brazil

## Introduction

Patients with septic shock die mainly due to refractory shock. Vasopressin is commonly used as an adjunct to catecholamines to support blood pressure in refractory septic shock, but its effect on mortality is unknown. We hypothesized that vasopressin as compared with norepinephrine would decrease mortality among cancer patients with septic shock.

## Methods

In this, randomized, double-blind trial, we assigned patients who had cancer and septic shock and needed a vasopressor to receive norepinephrine or vasopressin in addition to open-label vasopressors. All vasopressor infusions were titrated and tapered according to protocols to maintain a target blood pressure. The primary endpoint was the mortality rate 28 days after the start of infusions.

## Results

A total of 107 patients underwent randomization in this first part of trial, and were infused with the study drug (53 patients received vasopressin, and 54 norepinephrine), and were included in the analysis. There was no significant difference between the vasopressin and norepinephrine groups in the 28-day mortality rate (67.9 and 58.5%, respectively; *P *= 0.31). There were no significant differences in the overall rates of serious adverse events (5.3% and 5.5%, respectively; *P *= 1.00).

## Conclusion

Vasopressin did not reduce mortality rates as compared with norepinephrine among patients with cancer and septic shock who were treated with catecholamine vasopressors.

